# Improving medical certification of cause of death in Assiut University Children Hospital: an intervention study

**DOI:** 10.1186/s12913-024-11804-4

**Published:** 2024-11-29

**Authors:** Omaima El-Gibaly, Mohamed Gamil Mohamed Abo Elela, Yasser Farouk Abdel-Raheem Rizk, Shimaa Hosny Hassan Mahmoud

**Affiliations:** 1 Department of Public Health and Community Medicine, Faculty of Medicine, Assiut University, Assiut, 71515 Egypt; 2 Department of Pediatrics, Faculty of Medicine, Assiut University, Assiut, Egypt

**Keywords:** Improving, Cause of death, University hospital, Intervention, Egypt

## Abstract

**Introduction:**

Death certification is a health indicator and a public health surveillance tool. High-quality death certificate ensures reliability of mortality statistics used to direct the arranging of health-related programs and leading assessments of research and proper healthcare outcomes. The World Health Organization (WHO) puts Egypt in the group of ‘low quality’ death registration data. The aim of this study was to evaluate the impact of composite training and audit intervention on accurate completion of death notification forms (DNFs) in Assiut University Children Hospital (AUCH) that has an average monthly mortality of 120 children’s deaths.

**Methods:**

A Quasi-experimental study design was conducted among residents of AUCH. The intervention consisted of 1- Preparing training material with basic information on how to report causes of death according to WHO criteria and case scenarios extracted from the medical records of children who died at AUCH, 2- One hundred residents of the AUCH were trained in one day workshops in 4 groups, with a pre-post knowledge assessment questionnaire. 3- A weekly audit of a sample of 10–15 DNFs was done for six months with reporting of findings to quality assurance director of the hospital.

**Results:**

Eighty- nine physicians completed the pre-post knowledge assessment with significant increase in knowledge score after the intervention (15.7 ± 3.2 vs. 11.9 ± 2.8). There was a significant decrease in the errors of reporting on the DNFs. The main improvement was in decrease from 90 to 18% in reporting the mechanism of death, and significant decline in writing cause of death in Arabic language only.

**Conclusion:**

Accurate reporting of the medical cause of death can be achieved by educational intervention targeting physicians with institutionalizing of audit system for continuous quality improvement.

**Supplementary Information:**

The online version contains supplementary material available at 10.1186/s12913-024-11804-4.

## Introduction

Death certificate is an important legal and public health document issued by a hospital to prove the date, location, and cause of a person’s death. Data from death certificates is an essential component of national vital statistics. Death certificates are used by public health researchers for identification of the leading causes of death, disease outbreaks, surveillance of disease patterns, to determine public health funding and clinical research priorities [1]. 

Mortality data obtained from death certificates are essential for evaluating the success of public health programs, offering feed-back for future policy development and implementation, refining health planning and management, and determining priorities of health and medical research initiatives [2]. 

Moreover, tracking progress towards the Sustainable Development Goals (SDGs) will be unfeasible without dependable mortality data from civil registration and vital statistics (CRVS) systems. Cause-specific mortality data are required for 7 SDGs and 17 of corresponding indicators, with functioning CRVS systems being the optimal source [3]. 

Accurate certification of the cause of death (COD) is only feasible if physicians clearly understand the distinction between “cause” and “mechanism” of death, as well as “immediate” and “underlying” cause of death. The underlying COD is defined as “the disease, physical injury or intoxication that initiated the train of morbid events leading to death;”. whereas a mechanism of death refers to “a sequence of physiological or biochemical changes in the body, produced by the cause of death, that are incompatible with life” [4].

The legitimacy and effectiveness of mortality statistics is limited by inaccurate recording of COD. Validation studies and audits have demonstrated that heart disease is overrepresented as COD. This may be due to lack of training and failure to emphasize the importance of correct reporting of COD by hospital leaders. Although previous interventions as workshops and interactive training conducted have proved short-term improved COD reporting accuracy among trainees, their long-term changes as changes on population mortality statistics are still unassessed [5]. 

The national health database and the medico-legal investigations can be compromised by inaccuracy of the cause of death certification. A study at Benha University Hospitals was conducted to evaluate the errors of medical certification of cause of death and to recommend corrective procedures. The study reviewed death notification forms (DNFs) for all patients in 2014 and found errors in 100% of the DNFs [6]. 

The World Health Organization (WHO) classify Egypt as having ‘low quality’ death registration data, with completeness of “death certificates” < 31% and ill-defined codes appear on > 20% of registrations [7]. 

Errors in the data reported in death certificates can lead to the failure of prospective health policies. Major errors include listing multiple potential underlying causes of death, omission of the underlying cause of death and inappropriate sequencing of the causes of death. Minor errors involve reference of the mode or mechanism of death, overlooking the time interval between the onset of the cause and death, and incorrect use of abbreviations. These errors imply lack of quality of death certificates [8]. 

Although death certification is included in the curriculum of undergraduate medical students, its practical application is minimal. Few healthcare facilities or postgraduate training programs provide formal instruction on death certification, despite the importance of accurately recording information on death certificates [9]. 

In both high-income and low- to middle-income countries, the medical curriculum frequently focuses on legal or forensic aspects of death certification rather than highlighting its public health significance. This focusing can have a negative effect on the quality of information physicians record on COD and, subsequently, their diagnostic accuracy [10]. 

A medical officer initiates a death certificate in healthcare facilities, which is subsequently registered by a national civil registration system. Consequently, training medical doctors in death certification has become a crucial intervention to improve mortality statistics. Strategic investment in COD training performances will support long-term advances in the quality of cause of death data within CRVS systems, so expanding the efficacy of mortality data for health policy [11]. 

The International Form of Medical Certificate of Cause of Death (MCCD), commonly known as the death certificate, is recommended by the WHO for use in all countries. It offers a standardized framework for organizing clinical diagnoses for public health purposes. The form was last updated in 2016, after more than 50 years, as changes are rare due to the need for adjustments in national legislation and information systems [12]. 

Good-quality mortality statistics are best obtained by death certifications completed by a medically qualified resident physician. Death certification by residents is the “gold standard” for producing mortality data. Residents should understand importance, purposes, rules, guidelines, and expressions used in death certificates. More so in our setting where practice of performing autopsies is rare, death certificates have become an even more important source of data on mortality.

The present study aims:

- To evaluate the impact of structured training program for pediatric resident physicians at Assiut University Children’s Hospital on the accuracy of completing DNFs and reported cause of death.
- To audit death notification forms before and after training.
- To provide policy recommendations based on study findings to enhance the quality of death certification practices at a national level, ensuring alignment with international standards and the Sustainable Development Goals.

## Methods

### Study design

A Quasi-experimental study, pre- and post-intervention design was conducted among a single group of pediatric residents from Assiut University Children Hospital (AUCH).

### Setting and participants

All pediatric resident physicians (100 participants) at AUCH were the target for training of accurate completion of COD. The study setting is the largest children tertiary care hospital in Upper Egypt with 427 beds, receiving on average 1600 cases/month in the emergency room, admitted inpatients/month are on average 1400, and its outpatient clinics receives on average 6000 patients/month. It has 100 residents and an average of 30 newly appointed residents/year.

### Data sources

**Fig. 1:**
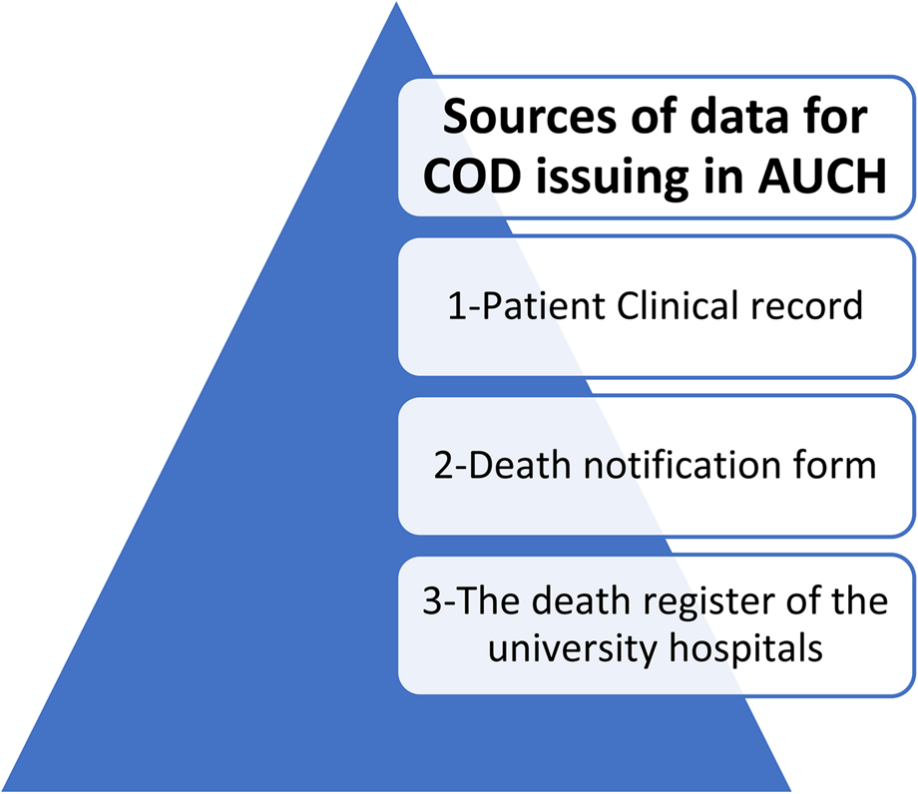
Sources of data for COD issuing in AUCH

There are 3 sources of data issued on the cause of death in AUCH (Fig. 1):

1. Patient Clinical record: section to notify death and report the cause of death.
2. Death notification form that is issued from the hospital and sent to the health office (ministry of health administration responsible for issuing official death certificate to the family of the deceased and burial permit).
3. The death register of the university hospitals that compiles all deaths and enters the data of the death notification forms of the hospital in one register.

- Cause of death as written in the clinical record of the patient is copied as it is in the DNF and in the register.

### Intervention

The study duration involved preparatory phase of 6 months and the actual intervention phase of another 6 months.

#### Preparatory phase

A multidisciplinary team of public health specialists, pediatricians of AUCH, administrators and quality assurance director of AUCH was assembled to do the following tasks:

1. Prepare training material and case scenarios from medical records of the children’s deaths at AUCH; the idea was to show how things were done and how they should be done.
2. Prepare lists of trainees and suggest the best training time and size for each batch.
3. To prepare the pre- and posttest assessment self-administered questionnaire.
4. To prepare checklist for the audit of the DNFs.
5. Examine the possibility of reviewing the monthly report of the death register of the AUCH, to identify improvements in reporting.
6. Preparing study protocol for submission to the Research Ethics Committee of the Assiut university Faculty of Medicine for approval.
7. As the cause of death section in the patient clinical record is the source of information for all other death sources (DNF and death register of the Assiut University Hospitals), the administration would explore the possibility to change the template in that section to be identical with WHO form ‘medical cause of death’, but administratively it would take too long.

*Preparation of training material* by public health specialists and pediatricians was done before conducting the study. Sixteen case scenarios were retrieved from the medical records of deceased children from 4 units: Neonatal Intensive Care Unit (NICU), Pediatric Intensive Care Unit (PICU), Emergency department and dehydration management units of the AUCH. The case scenarios described the history, clinical examination, investigations, treatment, complications and all medical procedures applied till death of the child.

#### Training phase

The training started in August 2023 and continued for 4 weeks. At the start of the training sessions, pretest was administered to residents to assess their knowledge on cause of death reporting and its significance.

### Training session details

#### Training

The session started with a 45-minute lecture and discussion on the following:

- Importance of accurate reporting of the cause of death.
- The responsibility of the resident physician to report cause of death and issuing DNFs.
- Components of the MCCD and how to complete it in both Arabic and English languages.
- Differentiation between cause and mechanism of death via examples.
- Differentiation between immediate and underlying cause of death.

*Practical training on case scenarios* retrieved from the AUCH health records. Residents in the training session were divided into groups of 5 physicians and asked to read the case scenario and think individually and then report the immediate and underlying cause of death in Arabic and English languages, time sequence of events, and any other comorbidities that contributed significantly to death.

After that, they discussed with the group the case scenarios and reported collectively on the immediate and underlying cause of death. Each case scenario took 5–10 min depending on their discussions and agreements. An assistant lecturer was facilitating the discussions and resolving any disagreements.

The training was provided by a public health specialist with attendance of a pediatrician to facilitate and guide the discussion of case scenarios.

The intervention was standardized across all sessions. Only the discussion of the case scenarios was tailored based on ongoing response from the participants on each training session.

*Four interactive workshops* of one day duration were conducted weekly for training of all resident physicians. Each workshop took about 4–5 h and involved about 25 participants.

### Data collection

#### Pre and post knowledge test

Introduction of the research team, facilitators and objective of the training together with administering the pretest to the participants was done. The Pretest was a closed-ended self-administered questionnaire of 18 questions. The questionnaire comprised an introduction to the training, outlined the study’s objectives, obtained participants’ consent, and collected details such as name, sex, and work duration. It also assessed knowledge regarding the completion of cause-of-death certificates. The test includes questions about when to report and who is responsible for reporting death, the template used, importance of death register, components of the international form of death certificate, how to complete medical COD section, differentiation between mechanism, immediate and underlying COD. The pre-posttest questionnaire was developed specially for this study. The reliability of the questionnaire was measured using Cronbach’s alpha which was equal to 0.5 (Supplementary file 1).

A pilot test -with a convenience sample of 10 house officers at AUCH- was organized, to evaluate clearness and effectiveness of the knowledge questionnaire. Based on the feedback from this pilot-testing, several revisions were made to the instrument before its official use.

*Post test* was administered via google forms to the residents 3 months after completing their training to assess their knowledge about COD reporting using the same pretest questionnaire.

Eighty-nine out of 100 residents completed the posttest assessment, resulting in 11 missing data points. To address this issue and mitigate potential attrition bias, we employed Complete Case Analysis, where only participants who completed the post-intervention assessment were included in the analysis. The missing data was assessed to be Missing Completely at Random (MCAR), which is said to be if the probability of being missing is the same for all cases. This effectively implies that causes of the missing data are unrelated to the data.

#### Pre and post audit of DNFs

*An audit of the DNFs at* AUCH was conducted before and after the training intervention. The audit involved a weekly review of a random sample of 10–15 DNFs, starting one month before the intervention and continuing once weekly for six months post intervention. Each weekly DNFs review was documented and reported to the quality assurance director. These reports identified pediatric residents who excelled and those who needed improvement, detailing errors encountered. The following data were collected and analyzed:

- Child name, age, department, file number and name of physician issuing the DNF.
- Date of admission and date of death.
- Time sequence.
- Mechanism of death.
- Other comorbidities and reporting in Arabic and English languages.

#### Reviewing AUCH death register

We reviewed the monthly report of the death register of the AUCH from August 2023 till February 2024 to extract the cause of death reported by residents. The register includes information on name, age, gender, date of admission and date of death, department and cause of death.

### Statistical analysis

The data was entered and analyzed using SPSS version 26. The pretest and posttest data were expressed as percentages, mean scores, and standard deviation (SD). Statistical significance was tested using paired T-test and McNemar test. P.value < 0.05 was considered as significant. The technical and medical errors in DNFs was depicted in percentages.

## Results

Table 1 displays demographic characteristics of the participants. The study included 100 pediatric resident physicians and assistant lecturers at AUCH. Only 89 completed the pretest-posttest procedure. The remaining 11 participants did not complete the post-test as they finished their residency period in the hospital, so they were excluded from the analysis. More than half of the participants were female (68.5%), most of them were resident physicians (87.6%) and nearly 60% of them have one to three years of work duration in the AUCH.

**Table 1:** Demographic characteristics of the participants

Characteristic	Number	Percentage
**Gender**		
Male	28	31.5
Female	61	68.5
**Job**		
Resident physician	78	87.6
Assistant lecturer	11	12.4
**Duration of work by months**		
Mean ± SD (Range)	20.78 ± 14.6 (4–60)	
**Duration of work by year**		
Less than one year	27	30.3
From one year to three years	53	59.6
More than three years	9	10.1

Table 2 shows the pretest posttest scores before and after the intervention. There was statistically significant difference before and after the intervention (P.value < 0.001) with higher mean score after the intervention (15.7 ± 3.2 vs. 11.9 ± 2.8). There was no significant difference in scores based on gender, but a significantly higher score was observed among resident physicians compared to assistant lecturers. Additionally, there was a significant increase in scores following the intervention, which was associated with an increase in work duration.

**Table 2:** Difference in answering knowledge test before and after the intervention

Characteristic	Pre-intervention	Post-intervention	*P*. value
**Knowledge score**	Mean ± SD		
	11.9 ± 2.8	15.7 ± 3.2	**< 0.001**
**Gender**			
Male	12 ± 2.9	15.4 ± 3.5	0.82
Female	11.8 ± 2.8	15.9 ± 3	0.39
**Job**			
Resident physician	11.5 ± 2.6	15.8 ± 2.9	**0.001**
Assistant lecturer	14.5 ± 3.4	15.4 ± 4.7	0.71
**Duration of work**	**One-way ANOVA**		
Less than one year	11.3 ± 1.9	16.2 ± 2.4	
From 1–3 years	11.72 ± 2.9	15.8 ± 3.3	
More than 3 years	14.78 ± 3.6	14.6 ± 4.5	
*F*. value	5.8	0.84	
*P*. value	**0.004**	0.43	

Table 3 shows the percentage of right answers before and after the intervention. Nearly the percentage of right answers were higher after the intervention than before the intervention in all the 18 questions with significant difference in 12 out of the 18 questions.

**Table 3:** The percentages of right answers before and after the intervention

Question	Percentage of right answers		*P* .value
	Pre-intervention	Post-intervention	
1-When should we report death occurrence?	41.6	64	**0.002**
2-Who is responsible for death reporting?	85.4	98.9	**0.002**
3-Which template is used for death reporting?	4.5	37.1	**< 0.001**
4-What version of ICD is used for Death reporting?	6.7	24.7	**< 0.001**
5-What is the importance of death registration?			
a-making statistical reports for mortality causes.	94.4	94.4	1
b-Measuring health level nationally and international.	74.2	83.1	**< 0.001**
c-Set health priorities.	51.7	73	**0.002**
d-Investigations of epidemic diseases.	84.3	86.5	0.82
e-Legal distribution of inheritance.	33.7	57.3	**0.001**
f-For medical research	55.1	70.8	**0.03**
6-Can physicians report such cases as cause of death?	3.4	77.5	**< 0.001**
7-How many parts are used to write cause of death?	16.9	87.6	0.48
8-How many lines in the 1st part of MCCD?	16.9	25.8	0.17
9-How many causes can be written in the 1st line of the 1st part of MCCD?	48.3	76.4	**< 0.001**
10-In which line the immediate cause of death is written?	70.8	86.5	**0.01**
11-What is written in the 1st and 2nd parts of MCCD?	25.8	66.3	**< 0.001**
12-What is written in the 2nd part of MCCD?	31.5	42.7	0.16
13-Can we write abbreviations in MCCD?	95.5	94.4	1
14-Is time sequence should be written between every line in MCCD?	18	58.4	**< 0.001**
15-What is the immediate cause of death?	82	89.9	0.2
16-What is the underlying cause of death?	78.7	78.7	1
17-What should be done in case of unnatural death cases?	70.8	86.5	**0.01**
18-What should be done in case of reporting death of a child before reporting birth?	51.7	91	**< 0.001**

Table 4 shows the distribution of audit of 372 DNFs from different units in the AUCH. Most of DNFs came from the hottest areas such as NICU (133), emergency department (114), PICU (62) and dehydration management unit (55). Time sequence was not reported at all until the end of the audit.

**Table 4:** Distribution of audit of DNFs by type of unit

Characteristic	Number (372)	Percentage
**Department of revised DNFs**		
NICU	133	35.8
PICU	62	16.7
Emergency	114	30.6
Dehydration management unit	55	14.8
Others	8	2.1
**Time sequence reported**		
No	372	`100

**Fig. 2:**
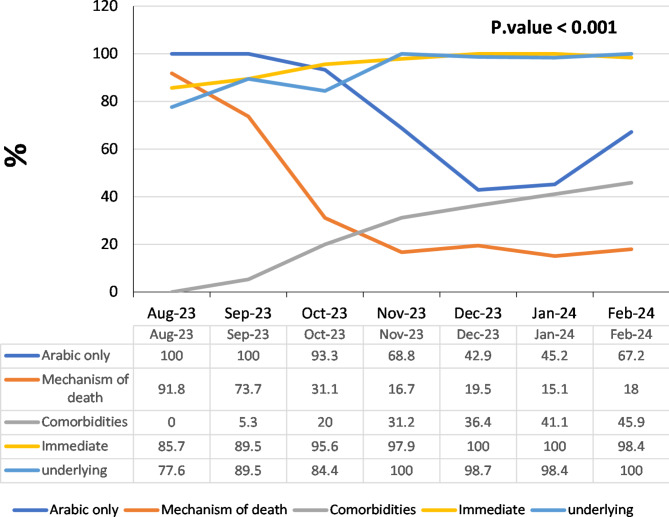
Improvement in reporting DNFs across time of audit

Figure 2 portrays the improvement in DNFs. There was significant improvement in issuing DNFs after the intervention. This is presented by significant decrease in writing the mechanism of death (from 91.8% before to only 18% after) and writing in Arabic language only (from 100% before 67.2% after). On the other hand, there was significant increase in mention of other comorbidities (from 0% before to only 45.9% after), reporting immediate cause of death (from 85.7% before to 98.4% after) and reporting underlying cause of death (from 77.6% before to 100% after).

**Fig. 3:**
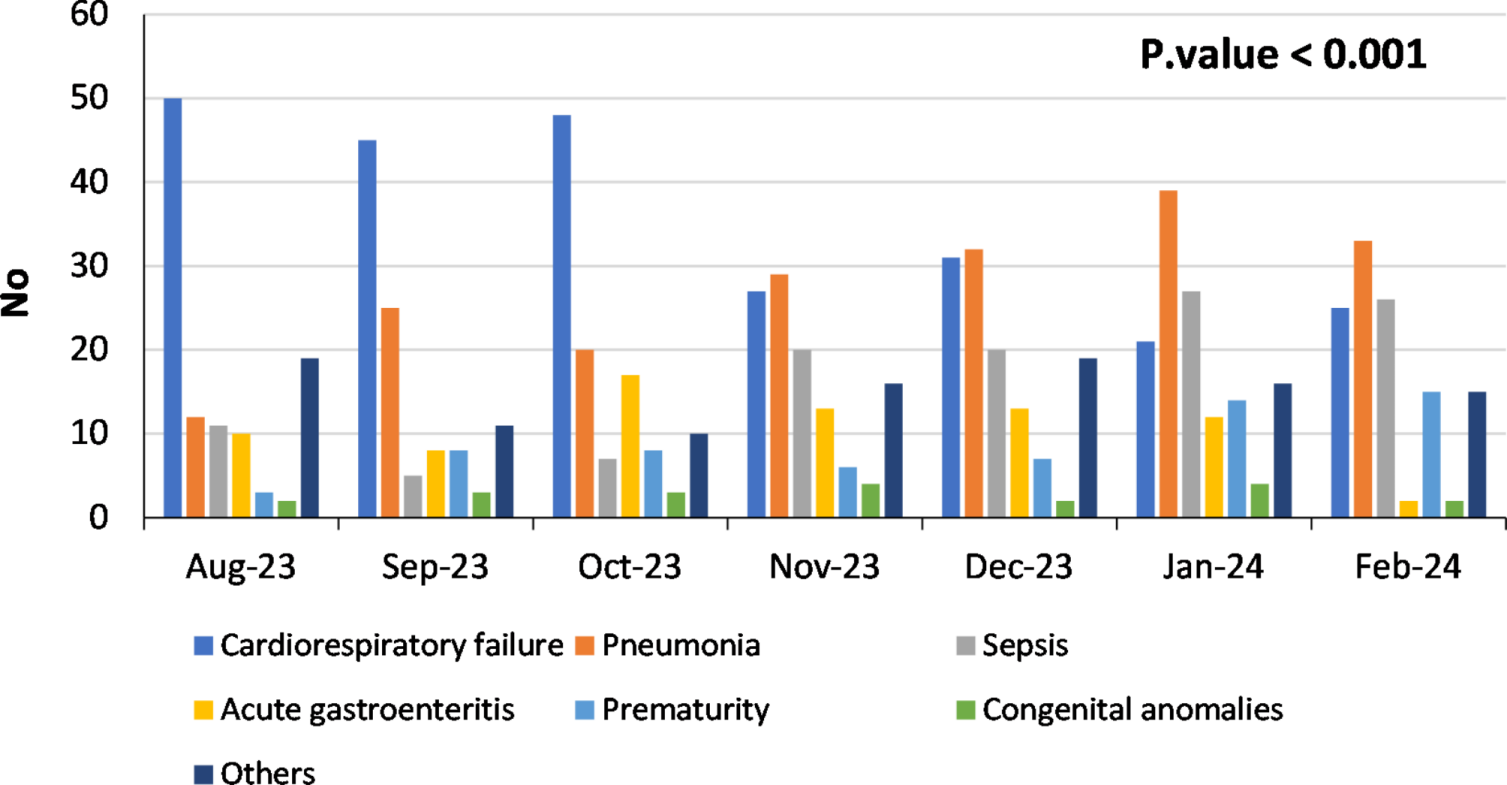
Cause of death from AUCH death register from August 2023 to February 2024

Figure 3 shows the cause of death as reported from AUCH death register. There was marked decrease in reporting and the mechanism of death “cardiorespiratory failure” as cause of death, from August 2023 till February 2024.

## Discussion

Medical certification of cause of death is a public health surveillance instrument from which cause-specific morbidity and mortality statistics are obtained. Poor quality of MCCD reporting will make this a wasted effort as the reliability of data will be doubtful. Training physicians regarding accurate reporting of DNFs is fundamental in avoiding such obstacles. Inaccurate mortality data have extensive international consequences further than the most apparent health care services, which should be emphasized.

Accurate and timely COD data are essential for tracking progress toward many of the SDGs and other health objectives [13]. Many of these health goals require COD and all-cause mortality data for the country, separated by age and sex [14]. To effectively measure progress towards SDGs, it is crucial that COD data are derived from a comprehensive and timely CRVS system, where each death’s cause is determined using the International Medical Certificate of Cause of Death, completed by a trained physician and following the certification and coding guidelines of the International Classification of Diseases (ICD-10) [12].

The present study was conducted to evaluate the impact of composite intervention on the knowledge and behaviour of physicians regarding accurate MCCD reporting. The current study revealed the effectiveness of training and audit interventions in increasing knowledge and accurate reporting of COD according to the guidelines of WHO.

This improvement was evident by significant increase in the knowledge about MCCD after the educational intervention with higher mean score after the intervention than before (15.7 ± 3.2 vs. 11.9 ± 2.8). Additionally, there was significant reduction in writing the mechanism of death instead of the COD (91.8% vs. 18%) and writing in Arabic language only instead of both Arabic and English (100% vs. 67.2%).

Several previous studied reported similar improvements in accuracy of death certification using different types of interventions. A systematic review of 24 selected interventions aimed to improving the quality of MCCD showed that training interventions significantly reduced the mistake rates among participants. Most interventions were carried out through interactive workshops, allowing participants to receive immediate training on site. These results highlight the possibility and importance of reinforcement of MCCD training, which will in turn increase the quality of mortality statistics [11]. 

Interactive workshops and printed instruction material were tried by Lakkireddy et al. and found that the interactive workshops were more effective [15]. Another study also used interactive workshop to allow participants to complete a death certificate case-scenario before and after workshop with decrease in number of errors after attending the training [16]. 

Both students, family physicians and interns showed improvement in professional completence using blended learning intervention which was consisted of seminar-workshop, on-line platform of basic information/documentation, preparation of death certificates based on clinical histories of real cases [17]. 

Using educational sessions, interactive workshops, and monthly audits for residents, Afzal et al., found that there was significant reduction in reporting mechanism with no underlying cause of death (60.0 vs. 14.6%) [18]. Significant decrease in reporting mechanism without underlying cause of death (13.5% v. 1%) was also reported by Pandya et al., following the educational intervention [9]. The most common error in the completion of death certificates before educational seminar was listing of the mechanism of death instead of the cause of death, 56.8% listed respiratory or cardiac arrest as the immediate cause of death compared to none after the educational intervention [19]. 

Three training strategies were used for assessment of MCCDs before and after training: (1) direct training of physicians (2) training of trainers and (3) implementation of an online and basic training. Reduction of incorrectly completed certificates was highest in the third strategy [10]. 

Improvement in knowledge about death certification was also reported by similar significant difference in pre- and postscores in previous studies [20, 21]. This improvement in knowledge could be translated into better mortality statistics for epidemiologic and legal purposes. Educational interventions are a good opportunity to bridge the gap in physicians’ training and practice.

Using a comprehensive educational intervention was effective in increasing the rate of accuracy in death certificates completion in a residency-based pediatric program, with significant difference in scores pre- and post-intervention [1]. 

Pre-post audit of death certificates revealed reduction in the number of certificates not meeting legal criteria as well as the number of mistakes and errors. This improvement has been achieved by simple educational measure delivered through three methods: (1) presenting findings anonymously in clinical meetings; (2) providing personalized performance data to each doctor; and (3) emphasizing the topic during the orientation of new physicians [22]. 

In the present study, there was no significant association between knowledge of MCCD and any of the demographic characteristics as gender, job position or work duration. This may be due to lack of training targeting death certification after start of residency. Therefore, each physician is issuing death certificate form his knowledge and as learned from his seniors. Bishwalata et al., also found no significance association between knowledge score and sociodemographic variables, as most of the study participants were also residents with a work experience of less than one year with no prior training on death certification [23].

In the present study, time sequence between underlying and the immediate cause of death, was not mentioned at all in all DNFs revised. Before the intervention, only 18% of participants answered “Yes” to the statement of “is time sequence should be written between every line in MCCD? “. This increased to 58.4% after the intervention. This highlighted the importance of educational intervention in increasing awareness about accurate completion of MCCD.

Auditing of DNFs in our setting revealed that death report was written in a paragraph form without any separate lines to write immediate or underlying cause of death and of course without any space or lines for time sequence. The standard WHO template of cause of death is not used in the medical records of patients in our setting.

The absence of the time interval was similarly the most common error reported in a study conducted at Benha University Hospitals, Egypt (96.5%) [6]. Madhao et al., also found that time interval lines were not printed in front of each line, which cause confusion in the minds and that is why many physicians opted to omit writing time sequence [24]. 

Although the present study finding was more than what was found in previous studies, but omission of ‘Time interval’ has always been found universal by nearly all researchers. as in Patil et al., (98.9%), Nojilana et al., (98.4%), Qaddumi et al., (97%), Pokable et al., (92%), Burger et al., (81.5%), Shantibala et al., (65.3%) and Pritt et al., (52%) [25–31]. 

A time sequence for each cause of death is critical to provide complete understanding of the cause of death and revealing underlying cause of death. The attending physician should pay great attention to this element wisely, because the chronology of events guarantees the correct sequence which in turn prevents the major error of improper sequencing [2]. 

Resident physicians have no experience in completion of death certificates and usually identify unacceptable cardiac and respiratory failure as the immediate cause of death. Madboly et al., stated that there was clear misunderstanding between the underlying cause of death and the method of death in 61.6% of revised DNFs [6]. These skills can be greatly improved with educational interventions with the interactive workshops being effective than routine lectures [15]. 

In addition, the significance of accurate reporting of cause of death is not sufficiently highlighted in medical curriculums. Most physicians are confused and assign the mechanism of death as the cause of death. The mechanism of death is a physiological disorder, or a biochemical disruption caused by the cause of death. For instance, pneumonia is a possible cause of death, but cardiac arrest or respiratory failure is a mechanism of death. Mechanism of death is not etiologically specific and so it should not appear on death certificates [32]. These differences are clearly stated in literature and textbooks and are lectured extensively to medical students. So that, cause of death should not be confused with the mechanism of death [33]. 

Another factor related to inaccuracy of death certificate is that the responsibility of death certification at teaching hospitals is usually assigned to the least experienced members of the team. This is usually accompanied with no proper training on the skill of writing a death certificate [9]. 

As observed in Philippines, training of physicians about precise completion of death certificates was proved to also have a longstanding effect on medical certification procedures. A large increase in error-free certificates after training, with a 41% fall in the average number of errors per certificate was reported [34]. 

### Strengths of the study

The current study utilized multiple approaches to assess the effectiveness of the training intervention, including pre-posttest evaluations, weekly audits with feedback to the quality assurance director, and a review of the AUCH death register post-intervention

Post-training assessments were conducted after 6 months, allowing the observed differences to reflect the long-term impact of the intervention. This method provides a more precise measure of the sustainability and effectiveness of the training compared to the more commonly used instant post-training assessments.

Additionally, all residents, and not just a sample of them, were trained on accurate completion of the COD, to avoid dilution of the impact of the training interventions by reviewing DNFs reported by residents who had not receive training.

### Limitation of the study

The study design was Quasi-experimental, purely pre-post without controls. The absence of a control group may limit the causality of the findings. So, future studies should consider a randomized controlled trial design to better establish causality. The section for reporting the COD in the patient clinical record could not be aligned with the WHO format during the study period due to administrative constraints.

The study was conducted in a single institution (Assiut University Children’s Hospital), which may limit the generalizability of the findings. Multi-center studies are recommended to enhance external validity. Although post-training assessments were conducted after six months, this period may still be insufficient to fully capture long-term retention of knowledge and changes in practice. Future research should include longer follow-up periods to determine the sustainability of the intervention’s impact.

Further research is needed to examine the relative effectiveness of different training approaches, such as online interventions versus those requiring direct interaction.

## Conclusion

The present study demonstrated that educational intervention significantly improves the accuracy of cause of death registration. The educational intervention provided had a significant increase in pediatric resident knowledge about death certification and a marked decrease in incorrect reporting mechanism of death rather than the cause of death.

### Recommendations

Medical curricula should be enhanced to include comprehensive instructions to medical students on accurate certification of COD and confirming consistent implementation of these courses. This is mainly the greatest cost-effective and enduring approach to enhance the quality of medical certification.

Since residency is a mandatory training period for all medical students, it is recommended that all resident physicians should receive training in accurate completing of MCCD.

Effective certification procedures mainly rely on residents’ attitudes toward the procedure, and the extent of monitoring, responsibility, and feedback regarding certification performance. Combining training with regular audits of MCCDs and providing consistent feedback to the physicians responsible for completing them enhances effectiveness.

Senior physicians should monitor daily DNFs issued by junior residents. Introduction of continued training session in the curriculum of postgraduate students, and hands on training every six months should be part of the requirements to finish residency.

**Fig. 4:**
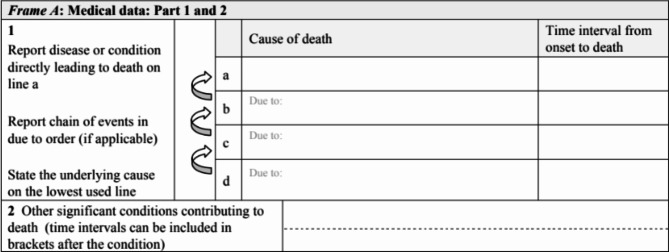
The WHO international form of medical certificate of cause death (MCCD) [12]

The WHO MCCD template Fig. 4 with its three parts, should be added to the patient medical records to encourage physicians to fill up the cause of death as required.

1. Part 1: report immediate and underlying cause of death.
2. Part 2: report other significant conditions contributing to death.
3. A column to record the time interval between each cause.

## Electronic supplementary material

Below is the link to the electronic supplementary material.

Supplementary Material 1.

## Data Availability

Data cannot be shared openly due to participant privacy but are available on reasonable request from the corresponding author.

## Abbreviations

WHO: World Health Organization
DNFs: Death notification forms
AUCH: Assiut University Children Hospital
SDGs: Sustainable Development Goals
CRVS: Civil registration and vital statistics
COD: Cause of death
MCCD: Medical Certificate of Cause of Death
NICU: Neonatal Intensive Care Unit
PICU: Pediatric Intensive Care Unit
SD: Standard deviation
