# Supplementary material for: Improving medical certification of cause of death in Assiut University Children Hospital: an intervention study
Source: BMC Health Serv Res. 2024 Nov 29;24:1506. doi: 10.1186/s12913-024-11804-4 (PMC11606018; doi:10.1186/s12913-024-11804-4)
Supplement: Supplementary file 1 — Supplementary Material 1. [file 12913_2024_11804_MOESM1_ESM.docx]

**Assiut University**

**Faculty of Medicine**

**Public Health and Community Medicine Department**

**Assiut University Children Hospital**

**Workshop for Improving Medical Certification of Cause of Death for Assiut University Children Hospital resident physicians: Pretest**

**Name**: ………………………………………..

**Gender**: 1-Male. 2-Female.

**Duration of work in Assiut University Children Hospital**: ………………….

Thank you for participating in the workshop. To achieve the training goals and to improve it in the future, we are conducting a pre-assessment test to identify the training needs of the residents in recording the medical cause of death. Please answer the following questions:

**1-When should we report death occurrence?**

1. 12 hours.
2. 24 hours.
3. 36 hours.
4. 48 hours.
5. I don’t know.
6. Uncertain.

**2-Who is responsible for death reporting?**

1. Nurses.
2. Physician.
3. Health Office employee.
4. Civil Registry employee.
5. I don’t know.
6. Uncertain.

**3-Which template is used for death reporting?**

1. 30 A.
2. 31 A.
3. 32 A.
4. 33 A.
5. I don’t know.
6. Uncertain.

**4-What version of ICD is used for Death reporting?**

1. 8^th^ version.
2. 9^th^ version.
3. 10^th^ version.
4. I don’t know.
5. Uncertain.

**5-What is the importance of death registration? Select all that apply:**

1. Making statistical reports for mortality causes.
2. Measuring health level nationally and internationally.
3. Set health priorities.
4. Investigations of epidemic diseases.
5. Legal distribution of inheritance.
6. For medical research

**6-Can physicians report such cases as cause of death?**

1. Shock.
2. Cardiac and respiratory arrest.
3. Cardiorespiratory failure.
4. None of the above.
5. I don’t know.
6. Uncertain.

**7-How many parts are used to write cause of death?**

1. One part.
2. Two parts.
3. Three parts.
4. Four parts.
5. I don’t know.
6. Uncertain.

**8-How many lines in the 1st part of MCCD?**

1. One.
2. Two.
3. Three,
4. Four,
5. I don’t know.
6. Uncertain.

**9-How many causes can be written in the 1st line of the 1st part of MCCD?**

1. Only one cause.
2. Two or more causes.
3. I don’t know.
4. Uncertain.

**10-In which line the immediate cause of death is written?**

1. Line a.
2. Line b.
3. Line c.
4. Line d.
5. I don’t know.
6. Uncertain.

**11-What is written in the 1st and 2nd parts of MCCD?**

1. Mechanism of death.
2. Cause of death.
3. Both mechanism and cause of death,
4. None of the above.
5. I don’t know.
6. Uncertain.

**12-What is written in the 2nd part of MCCD?**

1. Events directly related to the cause of death.
2. Events that contributed to the occurrence of death.
3. Used for statistical purposes.
4. A separate part not related to the cause of death.
5. I don’t know.
6. Uncertain

**13-Can we write abbreviations in MCCD?**

1. Yes.
2. No.
3. I don’t know.
4. Uncertain

**14-Is time sequence should be written between every line in MCCD?**

1. Yes.
2. No.
3. I don’t know.
4. Uncertain

**15-What is the immediate cause of death?**

1. Disease or injury that initiated the sequence of morbid events leading to death.
2. Last disease or injury leading to death.
3. I don’t know.
4. Uncertain

**16-What is the underlying cause of death?**

1. Disease or injury that initiated the sequence of morbid events leading to death.
2. Last disease or injury leading to death.
3. I don’t know.
4. Uncertain.

**17-What should be done in case of unnatural death cases?**

1. Conducting an external examination of the patient, writing the death certificate.
2. Informing the police department that the cause of death cannot be determined.
3. Calling for forensic medical examination.
4. Referring the patient to another physician for consultation.
5. I don’t know.
6. Uncertain.

**18-What should be done in case of reporting death of a child before reporting birth?**

1. A birth certificate and a death certificate are issued for the child.
2. Neither a birth certificate nor a death certificate is issued for the child.
3. A birth certificate is issued for the child, but no death certificate is issued.
4. A death certificate is issued for the child, but no birth certificate is issued.
5. I don’t know.
6. Uncertain.
